# Informal waste pickers and non-motorised transport: A mini-review of contemporary literature

**DOI:** 10.1177/0734242X251361730

**Published:** 2025-08-27

**Authors:** Pascaline Wibin, Marc Kalina

**Affiliations:** 1 Department of Environmental Systems Science (USYS), ETH Zurich, Zurich, Switzerland; 2 Department of Mechanical and Process Engineering (MAVT), ETH Zurich, Zurich, Switzerland; 3 Centre for Interdisciplinary Studies of Children, Families and Society (CISCFS), University of the Western Cape, Cape Town, South Africa

**Keywords:** Informal waste pickers, non-motorised transport, municipal solid waste, recycling, circular economy

## Abstract

Informal waste pickers (IWPs) – individuals who collect, sort and sell recyclable materials outside formal waste management systems – play a critical role in urban waste recycling and landfill reduction. Despite their vital contributions, their reliance on non-motorised transport (NMT) – such as pushcarts, bicycles and wheelbarrows – remains underexplored. This mini-review assesses the relationship between IWPs and NMT, highlighting key challenges, opportunities and research needs. The findings reveal that NMT enhances the mobility and livelihoods of IWPs, enabling them to transport recyclables over long distances and challenging terrains. However, inadequate infrastructure, including a lack of storage facilities, dedicated pathways and poorly designed vehicles, hinders their productivity and safety. Social stigma and marginalisation further exacerbate these challenges, as IWPs and their vehicles are often viewed negatively. Gender disparities also persist, with women facing additional barriers in accessing and using NMT due to societal norms and physical constraints. Current literature predominantly focuses on qualitative insights, leaving key quantitative gaps. Data on the economic benefits of NMT, the long-term health impacts of its use and the role of infrastructure improvements in optimising outcomes are scarce. Research is largely region-specific, with limited cross-regional synthesis or multi-lingual reviews. There is also a need for contextualised interventions and ultra-local research tailored to the specific challenges and opportunities faced by IWPs in diverse environments. This mini-review article calls for changes, including inclusive urban planning, policy recognition and community-driven solutions. Bridging research gaps through evidence-based and context-sensitive interventions is critical for enhancing IWPs’ livelihoods and advancing sustainable waste management practices globally.

## Introduction

Informal waste pickers (IWPs) play a crucial role in global waste management systems. Operating alongside, or in the absence of, formal waste collection systems, IWPs salvage reusable or recyclable materials thrown away by others, either to sell or for personal consumption or use (Binion and Gutberlet, 2012; Coletto and Bisschop, 2017; Kasinja and Tilley, 2018). Collecting at landfill or on the streets, IWPs are a familiar sight in many cities across the globe, particularly within the Global South, where through their waste diversion and recycling efforts they are essential to municipal waste management systems and national recycling economies (Samson et al., 2022).

By diverting recyclable materials that might otherwise be landfilled, IWPs save municipal systems immense sums per year in potential landfill costs, while contributing to reducing greenhouse gas emissions, decreasing demand for non-renewable raw materials, all while supporting an estimated 15–20 million livelihoods globally (Coletto and Bisschop, 2017). At a country or city-level, the importance of these contributions are even more apparent, with IWPs responsible, for instance, for recovering 50–80% of India’s plastic waste (Gutberlet, 2023; Nandy et al., 2015), managing one-third of Cairo’s waste while recycling 80% of it (Gutberlet, 2023; Linzner and Lange, 2013), or collecting up to 50% of waste in Pakistan and the Philippines (Liang et al., 2021), to highlight a few examples. Nonetheless, despite their importance, IWPs have been systematically delegitimised: treated as competition or even criminals by the state, and stigmatised by the communities that they work within (Aparcana, 2017). However, over the past two decades, there has been growing consensus and increased interest within waste management academic discourse over the value that IWPs provide to both individual livelihoods, national recycling economies and to municipal waste systems, and as a result, informal recyclers have increasingly been discussed within contemporary waste management literature as an asset rather than a nuisance.

IWPs are referred to by names as diverse as their contributions. From ‘canners’ or ‘binners’ in Canada (Sholanke and Gutberlet, 2020), ‘reclaimers’ in South Africa (da Silva et al., 2019), ‘catadores’ in Brazil (Silva de Souza Lima and Mancini, 2017), ‘zabaleen’ in Egypt (Fahmi, 2005) or less charitably, ‘scavengers’ or ‘rag-pickers’ in many locations across the globe (Das et al., 2023), IWPs are generally identified through their relationship to waste. Note that the term ‘waste picker’ was adopted at the First World Conference of Waste Pickers in Bogota, Colombia, in 2008 as a standardised moniker to facilitate global networking and to combat stigmatising alternatives (Waste Pickers Without Frontiers, 2008). Yet, although IWPs are mostly commonly defined by the public in relation to waste, other names common in some countries, such as cart-pusher or barrow-boy in Nigeria (Ebekozien et al., 2024; Fadugba et al., 2024) capture waste picker’s most visible relationship to the predominantly non-motorised vehicles they use to transport waste across cities. Non-motorised transport (NMT) refers to all forms of transport that do not rely on an engine or motor for movement. Waste pickers most often rely on their feet for transport, walking great distances to collect and deliver recyclables, but many also utilise various non-motorised vehicles to facilitate their work. The types of vehicles used vary by context and opportunity and are as diverse as the names attached to IWPs across the globe, including bicycles, rickshaws, pushcarts, shopping carts and wheelbarrows, to name a few (Rouse and Ali, 2002). Non-motorised vehicles serve a transport gap for informal workers, facilitating material transport where motorised vehicles are inaccessible, infeasible or otherwise out of reach. Contemporary research on informal recyclers frequently touches upon the role of NMT in IWP lives and livelihoods, for instance, by highlighting NMT’s role in enabling IWPs to transport more waste over longer distances (Brem et al., 2020; Viljoen et al., 2018), influencing their social status by either stigmatising or empowering them (Brem et al., 2020), and their impact on working conditions for IWPs (Shah et al., 2019). Although we know that the relationship between IWPs and NMT is important, and a daily factor within worker’s lives, the depth and scope of those relationships remain poorly understood. Considering growing interest in inclusive circular economy approaches and sustainable urban waste systems, a clearer understanding of this relationship is essential to inform future research, policy and design.

This mini-review therefore seeks to answer a central question: what is currently known about the relationship between IWPs and NMT, and where do significant knowledge gaps remain? Drawing on a broad survey of both contemporary and foundational IWP literature, it seeks to identify and assess cross-cutting themes, pinpoint gaps in current understandings and highlight areas in need of further study. The goal is to describe what we, as scholars and practitioners, know and do not know about IWPs relationships with NMT and to outline a future research agenda which shores up gaps within our current knowledge. Our findings highlight a lack of specific studies, and peer-reviewed work in particular, on how IWPs use, relate to, and leverage NMT within their lives and livelihoods. Although NMT is frequently mentioned in passing, or linked to IWPs, within literature, those relationships are rarely explored in depth, signify a critical knowledge deficit which may impede policy or programmatic efforts towards improving IWPs’ working conditions and livelihoods. The themes that do emerge, suggest that NMT has strong, positive, impacts on IWPs livelihoods and sense of identity, while underscoring the multifaceted importance of NMT design in impacting social perceptions, safety, practicality and health. Challenges around NMT centre on inadequate policy, planning and infrastructure to support safe movement, storage and maintenance. Furthermore, although IWPs and NMT remain marginalised in most urban contexts in which they work, limited programmatic interventions, which address practical and policy barriers, have shown success in progressing systemic change, whereas successful community-driven initiatives have enhanced systemic change, while facilitating broader waste picker integration objectives. Key knowledge gaps centre on the importance of vehicle design, the role of inclusive public planning and supporting infrastructure, social status and gender inequalities associated with these vehicles, as well as their certification and legal recognition in public spaces, while highlighting the need for holistic and relational responses which support waste pickers rights to waste and the city, while incorporating all actors along the waste value chain. This comprehensive approach is essential for promoting inclusive and sustainable waste management practices and improving the lives and livelihoods of IWPs worldwide.

## Methodology

The aim of this review is to survey IWP literature to draw out current understandings on the relationship between IWPs and NMT while identify knowledge gaps which can serve as the focus for a future research agenda. Given the interpretive nature of this aim, and the scale and breadth of literature surveyed, a narrative method was adopted, albeit with a systematic approach and defined search and exclusion criteria guided by the Preferred Reporting Items for Systematic Reviews and Meta-Analyses (PRISMA) guidelines (Gregory and Denniss, 2018). The PRISMA checklist informed the structuring of the search, screening and inclusion process (Page et al., 2021). In particular, the steps of identification (systematic search), screening (titles and abstracts), eligibility (full-text assessment) and inclusion (final selection) were applied. Utilising these guidelines, a systematic search using Boolean terms was conducted in Google Scholar. Google Scholar was selected due to its accessibility, as well as the topic’s specific and narrow scope, which limited results in more specialised databases.

Search terms were developed through an iterative process, considering the number of hits and their quality. Multiple search terms were combined to produce different iterations and combinations of IWP and NMT, such as ‘informal waste picker’, ‘recycler’, ‘binner’, ‘scavenger’ or ‘reclaimer’ with ‘wheelbarrow’, ‘non-motorised transport’, ‘donkey-cart’, ‘cart’ or ‘pushcart’. This process retrieved over 900 articles, the abstracts and title of which were screened for keyword combinations related to IWPs and NMT. If absent, the full text was reviewed to assess whether NMT use by IWPs was explicitly discussed or only referenced in passing. Articles were retained if they included multiple references to NMT or dedicated sections on its role. Peer-reviewed and grey-literature were considered, including theses and dissertations; however, efforts were made to minimise these contributions, while grey-literature was specifically scrutinised for quality and legitimacy. In addition, peer-reviewed journal articles were assessed for quality. Specifically, articles were only included if the publishing journal was clearly legitimate, indexed and not flagged by any nationals or watchdogs. Articles from journals or publishers that appear in Beall’s Predatory Journals and Publishers list or Cabell’s Predatory Reports were excluded. The initial screening and full-text assessments were conducted by the lead author, with a subset cross-checked by the second author to support consistency and reduce selection bias. This had led to the inclusion 81 articles within the review, dating from 2000 to 2023.

Reflecting the interdisciplinary nature of the topic, these article span multiple disciplines and their associated methodologies, including engineering, geography, sociology, medicine and law. Only English literature was included. This exclusion likely limits the scope and representativeness of the findings, particularly given that significant research on informal waste picking – especially from Latin America, where the practice is widespread – is published in Spanish and Portuguese. As a result, important regional experiences, innovations, or contextual nuances may not be reflected in this review. This language bias could skew the geographic distribution of evidence and underrepresent community-driven initiatives or locally relevant interventions. Future reviews should prioritise multilingual inclusion to improve comprehensiveness, reduce replication of research, and strengthen the applicability of findings across global contexts.

## The literature

The 81 articles included within the review were read, with themes identified and coded in Taguette. As noted, the identified literature spans a broad and diverse range of disciplines, and although a number of clear themes were discernible, clear trends regarding the quality and scope of the literature were also evident. Firstly, is the preponderance of so-called grey literature, with only 60% (49 articles) of peer-reviewed literature. Secondly, although, the work spanned several disciplines, primary studies identified were overwhelmingly qualitative, with very little quantitative research present. A geographical imbalance in the distribution of publications was also evident, with work from Canada and, to a lesser extent, the United States, preponderant amongst contributions from the Global North, whereas work from South Asia countries, Brazil and South Africa, represent the overwhelming majority of contributions from Global South countries, with limited mention of other countries such as Colombia, China or Kenya, and little to no representation of the rest. This imbalance may be indicative of a research gap, but may also be rooted in this review’s focus on English language research which may have excluded contributions from other research contexts.

Included studies were found to fall within six main themes, with distinct sub-themes within. The following sections reflect this characterisation: NMT typologies and design, acquisition and ownership, NMT infrastructures, health, social factors and NMT initiatives. Finally, although the findings were thematically organised, it is important to mention that there are instances of overlapping themes leading to some repetitions. For instance, a more efficiently and ergonomically designed cart will increase livelihood and reduce health impacts, while a certain urban layout may enhance gender inequalities. The studies highlighted in the sections below were selected as those studies most relevant to the relationship between IWPs and NMT.

### NMT typologies and design

#### Types of NMT

**Figure 1.**
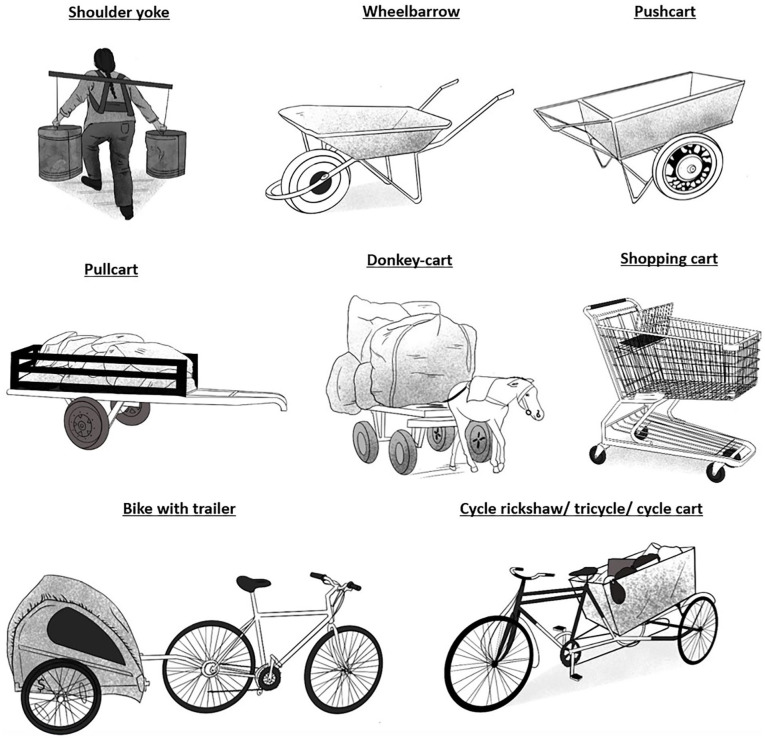
Typologies of non-motorised transport.

Different forms of NMT are used depending on different contexts and criteria such as location, price, gender and traditions (see Figure 1). The most used vehicles are carts of any kind, with two, three or four wheels, such as pushcarts, pull carts or shopping-carts (Ahn et al., 2020; Brem et al., 2020; Gutberlet et al., 2009; Linzner and Lange, 2013; Orlins and Guan, 2016; Rendon et al., 2021; Thakur et al., 2018). Cycle carts, known as cycle rickshaws or tricycles are seen where the topography enables its use, such as flat ground. They usually allow for more load than wheelbarrows or carts (Calafate-Faria, 2013; Das et al., 2023; Furedy, 1993; Imam et al., 2008; Sholanke and Gutberlet, 2022; Thakur et al., 2018; Wilson et al., 2009; Zia et al., 2008). Many recyclers in Canada and United States ride bikes with big bags on their shoulders, or extra carts attached behind it (Elliott, 2019; Steinmann, 2020; Steinmann and Wilson, 2022, 2023). Wheelbarrows are usually cheaper than carts as they have only one wheel and are more basic but can transport only small loads (Fidelis et al., 2023; Imam et al., 2008; Makina, 2020; Rouse and Ali, 2002; Uteng, 2011; Wilson et al., 2009).

Horse-carts and donkey-carts are also utilised across contexts as diverse as Brazil, Nigeria and Pakistan. Although the maintenance of the animal represents a considerable cost, they can transport up to 1 tonne, which is double than the biggest carts (Calafate-Faria, 2013; Fahmi, 2005; Nzeadibe, 2014; O’Hare, 2020; Rouse and Ali, 2002; Shah et al., 2019). Finally, some literature mention yokes and simple back frames, which are practical for steep hills or where wheeled vehicles cannot be used (Mitchell, 2008; Rouse and Ali, 2002).

#### Design considerations

Due to variations in needs among countries and individuals, designing a vehicle that accommodates all IWPs across all contexts is impossible, and design considerations must be determined by local needs and challenges. As the literature identified, in numerous cases, the equipment used is inadequate, and the design is dictated by availability and prices of material (Brem et al., 2020; Rouse and Ali, 2002), resulting in challenging mobility and usage issues such as unprotected cartons that become heavier during rainy conditions (Bala et al., 2023; Brem et al., 2020; Calafate-Faria, 2013; Elliott, 2019; Steinmann, 2020). Modification and adaptation of vehicles to suit local contexts and challenges appears common, and various additional features have been observed on different vehicles, including braking pads (Calafate-Faria, 2013), rear mirrors (Calafate-Faria, 2013), openings for sleeping accommodation (Calafate-Faria, 2013; Gutberlet et al., 2009), hooks (Calafate-Faria, 2013; Rouse and Ali, 2002), weather protection (Calafate-Faria, 2013; Elliott, 2019), changes in handle materials (Elliott, 2019; Samson et al., 2020), removal of pedals (Rouse and Ali, 2002), manual scales (Ahmed et al., 2022), reflectors (Ahn et al., 2020), waste segregation systems (Rouse and Ali, 2002) or small compactors (Rouse and Ali, 2002). Design considerations are crucial for recyclers’ income and health as they increase the vehicle’s efficiency and capacity (Brem et al., 2020). Design should also depend on the types of waste being carried, and be determined by factors such as the density, corrosiveness and abrasiveness of the waste (Rouse and Ali, 2002). Easy emptying should be facilitated, as it is often impossible to empty vehicles like carts, tricycles or donkey-carts alone. Additionally, such design considerations can address gender disparities, as women may face difficulties due to lower physical strength, making them more dependent and vulnerable in the pushing and emptying process (Calafate-Faria, 2013; Rouse and Ali, 2002; Valente et al., 2021). Finally, the design of vehicles should involve collaboration among academics, manufacturers and recyclers. Neglecting the involvement of the latter could result in inefficient or unusable vehicles (Brem et al., 2020; Samson et al., 2020).

### Vehicle acquisition and ownership

#### Acquisition

IWPs acquire their vehicles in diverse ways, including through private employers (Elliott, 2019; Rouse and Ali, 2002), local authorities (Mitchell, 2008; Rouse and Ali, 2002; Valente et al., 2021), deposit owners (Rouse and Ali, 2002), community groups (Bala et al., 2023; Rouse and Ali, 2002; Silva de Souza Lima and Mancini, 2017; Steinmann, 2020; Viljoen, 2014), build it themselves with available material (Bala et al., 2023; Gutberlet and Baeder, 2008; Rouse and Ali, 2002; Steinmann, 2020), buy it from people they know (Viljoen, 2014) or even steal it (Viljoen, 2014; Yu et al., 2020). Finding suitable tools for work, including vehicles, was identified as challenging for recyclers (Elliott, 2019; Gutberlet and Baeder, 2008; Gutberlet et al., 2009; Samson et al., 2020), and even for those who find one, there is a trade-off between cost, availability and efficiency (Elliott, 2019). Possessing one’s own equipment is a key factor in achieving independence, particularly in dealing with middlemen (Calafate-Faria, 2013; Coletto and Bisschop, 2017; Hayami et al., 2006). In Vancouver, the Downtown Eastside Street Market (DTES Street Market) serves as a hub where IWPs can purchase bikes and trailers. Additionally, recyclers can access materials from community bike shops (Elliott, 2019; Steinmann and Wilson, 2023, 2023).

Furthermore, although our has been on recyclers using forms of NMT, it is worth noting that a minority of IWPs use motorised vehicles for their operations. These vehicles, often vans, trucks or motorised rickshaws, have been found to offer several advantages over NMT in some contexts, including greater collection capacity (Calafate-Faria, 2013), greater distance covered (Calafate-Faria, 2013; Rendon, 2020; Rendon et al., 2021), easier access to NMT restricted areas (Chen, 2016) and a reduced risk of musculoskeletal problems (Navarrete-Hernandez and Navarrete-Hernandez, 2018). In Canada, for example, ownership of such a vehicle allows access to recycling centres and the sale of materials at higher prices (Steinmann, 2020). Furthermore, for IWPs, owning a motorised vehicle symbolises a rise in the social hierarchy (Chen, 2016; Goeiman, 2020; Hildebrand, 2016). As a result, many IWPs strive to accumulate savings for the purchase of a motorised vehicle in order to improve their livelihoods while remaining integrated into the informal market (Calafate-Faria, 2013; Navarrete-Hernandez and Navarrete-Hernandez, 2018; Omokaro and Taipale, 2018; Uddin and Gutberlet, 2018).

#### Vehicle maintenance and storage

Despite their perceived and potential impacts, NMT vehicles are not without cost to IWPs and require constant effort and resources in order to be maintained and stored securely. Vehicles used by recyclers are typically constructed with basic mechanics and inexpensive materials, allowing users to handle maintenance personally. When it is not possible, roadside workshops or large municipal manufacturing units may offer repairs, usually under the responsibility of the employers. Unfortunately, it is noted that maintenance is often neglected when a vehicle is damaged. This can be attributed to factors like a lack of knowledge, money or time on the part of the IWP, prolonged repair times leading to income loss, employer/ non-governmental organisation’s (NGO’s) irresponsibility or frequent breakdowns making repairs impractical (Rouse and Ali, 2002). If preventive maintenance would be advisable, this practice is often overlooked (Rouse and Ali, 2002). In Vancouver, IWPs demonstrate skills in bike repairs and have access to community bike shops that provide them with bike parts and repair services in exchange for volunteer hours or deferred payment (Steinmann, 2020).

When not in use, vehicles must be securely stored to prevent theft, which is reported to be a difficult task (Elliott, 2019; Rouse and Ali, 2002; Wittmer and Parizeau, 2018). While employers may sometimes bear this responsibility, it typically falls on the user (Rouse and Ali, 2002). Many vehicles are just chained somewhere on the street, but this exposes them to adverse weather conditions, shortening their lifespan (Calafate-Faria, 2013; Elliott, 2019; Rouse and Ali, 2002; Steinmann, 2020). Several storage-options are available: some recyclers store vehicles in a government provided place (Calafate-Faria, 2013; Rouse and Ali, 2002), others in their own accommodation if they have enough space and good access (Elliott, 2019; Rouse and Ali, 2002; Steinmann, 2020) or some might pay for a private secured place (Rouse and Ali, 2002). In Vancouver, some individuals even sleep on their bikes to safeguard them (Elliott, 2019; Steinmann, 2020). Nonetheless, theft incidents were reported in all cases, adversely affecting recyclers’ efficiency in waste collection (Bala et al., 2023; Calafate-Faria, 2013; Chen, 2016; Elliott, 2019; Makina, 2020; Rouse and Ali, 2002; Schenck and Blaauw, 2011; Steinmann, 2020; Steinmann and Wilson, 2022). Ensuring the security of their work tools, including vehicles, emerged as one of the key challenges faced by recyclers in North-Central Surrey (Elliott, 2019).

### NMT infrastructures

#### Policy infrastructure

It was found, that within most cities, urban policy does not consider the needs of NMT users (Department of Environmental Affairs, n.d.; Gwala, 2007; Mitullah and Opiyo, 2012; Sing, 2018). Bicycle-related laws and infrastructure primarily target recreational and commuting cyclists, neglecting the needs of recyclers and sometimes even hindering their activities, such as prohibition from cycling on sidewalks (Steinmann, 2020; Steinmann and Wilson, 2022, 2023). Aside from bicycles, in many cities, transport policies prioritise private car owners, limiting the accessibility and viability of NMT for recyclers (Mbah et al., 2019; Uteng, 2011). For example, bans on certain types of transport, such as cycle rickshaws in Delhi (Rouse and Ali, 2002; Uteng, 2011) or animal-drawn vehicles in Colombia (Sing, 2018), severely restricted the mobility and livelihoods of IWPs. Furthermore, bylaws governing the design of waste collection vehicles in Johannesburg included cart features unsuitable for recyclers (Koen and Fourie, 2023). In addition, regulations in several countries, including Canada and the United States, restrict recyclers’ access to recycling centres by banning carts and bicycles or by implementing strict regulations (Chikowore and Kerr, 2020; Nandy et al., 2015; Rendon, 2020; Steinmann, 2020). Moreover, many recyclers are denied the use of the bus if they carry their collection in bags, prohibiting them to access the recycling centre even if they do not have a cart or bike (Chikowore and Kerr, 2020).

IWPs are often powerless in the face of government regulations, adding to the challenges of utilising NMT within urban spaces and increasing their own vulnerability to law enforcement entities (Rouse and Ali, 2002). Disenfranchising or non-inclusive policy has contributed to altercations with the police, and seizures were recorded for various reasons, including: usage of a grocery cart (Elliott, 2019; Gutberlet et al., 2009; Imam et al., 2008), recycling in a prohibited area (Hildebrand, 2016) or non-respect of bylaws (Koen and Fourie, 2023; Steinmann, 2020). In particular, the type of vehicle used can attract unwanted attention from the authorities, resulting in more frequent arrests or seizures (Chen, 2016; Hildebrand, 2016; Steinmann, 2020).

#### Physical infrastructure

Urban design, and presence (or lack) of enabling infrastructure significantly impacts the work of recyclers, and their ability to navigate the city with NMT. Many urban areas are primarily structured for motorised vehicles, such as cars and buses (Calafate-Faria, 2013; Department of Environmental Affairs, n.d.; Makina, 2020; Uteng, 2011). Recyclers using pushcarts or bicycles often navigate through traffic, risking injury and enduring constant harassment due to their presence on the streets. Furthermore, when there is infrastructure provided for NMT, IWPs are often excluded. For instance, IWPs can be fined or have their vehicle confiscated for using sidewalks, which is prohibited in many cities (Gutberlet and Baeder, 2008; Melo, 2019; Rendon, 2020; Schenck and Blaauw, 2011; Uteng, 2011). In Canada, the location and type of bike infrastructures, such as too thin bike lanes at impractical locations, seems to contribute to the marginalisation of recyclers as they are designed for leisure or commuting cyclists (Steinmann, 2020; Steinmann and Wilson, 2022).

Road conditions further impact transportation choices. In areas with holes and poor surface quality, vehicles with smaller wheels become less viable (Ahn et al., 2020; Elliott, 2019; Makina, 2020; Omokaro and Taipale, 2018; Rouse and Ali, 2002). Conversely, narrow streets may prevent formal waste collection trucks from entering, offering opportunities for informal recyclers with smaller vehicles to perform the collection (Malik, 2023; Nesheim et al., 2023). Hilly terrain poses challenges, especially for women or disabled people, as it requires significant strength to push carts uphill or control their descent (Rouse and Ali, 2002). Furthermore, it is reported that transfer points and waste dealers are often situated far from collection routes or in areas difficult to access. As a result, some recyclers may need to rent motorised vehicles or bicycles, or cycle on major roads or sidewalks. Moreover, they tend to overload their vehicles to minimise the number of trips, which can lead to damage and increase maintenance costs (Chen, 2016; Rouse and Ali, 2002; Samson, 2020; Steinmann and Wilson, 2023).

Housing density is another critical factor for recyclers, as lower density areas necessitate longer travel distances (Kasinja and Tilley, 2018; Rendon, 2020; Rouse and Ali, 2002). This situation leads many IWPs to work in the city centre, sometimes requiring them to stay overnight (Parizeau, 2011, 2013; Schenck and Blaauw, 2011). For those who reside far from their collection areas, using NMT while commuting to work can pose significant challenges, especially when utilising public transport. For instance, carrying a shopping cart becomes impractical during long bus rides, and trash bags carried by recyclers are often prohibited on buses (Chikowore and Kerr, 2020; Elliott, 2019). Those using subways, buses or simply bicycling are therefore more likely to store or rent carts near their workplaces (Parizeau, 2013). Although seatless trains in Buenos Aires allowed recyclers to travel with carts, they ceased operation in 2007 and were replaced with trucks running at inconvenient schedules and allowing only cardboard and paper carts (Parizeau, 2011). Due to these challenges, many recyclers, unable to return home daily, end up sleeping in the streets in their carts or in informal settlements, or are unable to work (Calafate-Faria, 2013; Chen, 2016; Parizeau, 2011, 2013; Schenck and Blaauw, 2011).

### Health

The survey suggests numerous linkages between NMT and IWP health. Firstly, the vehicle can be seen as a health or mobility asset: recyclers who carry bags, often on their heads, face the risk of hand and facial injuries (Rouse and Ali, 2002). Moreover, bicycling is considered by IWPs to maintain good health through exercise and serves as a helpful mobility aid. Some IWPs, who cannot walk for extended periods, find cycling to be a viable option (Steinmann and Wilson, 2022). For others, bicycling might be too challenging, but grocery carts become useful as one can lean on them for support (Elliott, 2019). However, the use of a badly designed vehicle has been found to contribute to numerous health issues including musculoskeletal injuries (Ahn et al., 2020; Bala et al., 2023; Binion and Gutberlet, 2012; Brem et al., 2020; Gutberlet and Baeder, 2008), which have been successfully addressed through ergonomic interventions such as height adjustment (Ahn et al., 2020; Elliott, 2019; Rouse and Ali, 2002). In Canada, literature mentions anxiety associated with the possession of a vehicle due to thefts (Elliott, 2019). Other risks include cuts and nerve damage on hands due to inadequate cart handles (Brem et al., 2020; Elliott, 2019; Rouse and Ali, 2002), cuts and diseases from touching waste if the cart cannot be lifted to be emptied (Rouse and Ali, 2002), injuries on trains caused by moving carts in Buenos Aires (Parizeau, 2013) and accidents in traffic (Ahn et al., 2020; Binion and Gutberlet, 2012; Gutberlet and Baeder, 2008; Makina, 2020; Melo, 2019; Steinmann and Wilson, 2022). Visibility in traffic-related accidents has been improved with reflectors on carts and clothes, along with encouragement to adhere to traffic rules (Ahn et al., 2020; Gutberlet and Baeder, 2008).

### Social factors

#### Stigma

Despite their contributions, IWPs face intense social stigma, both from the state and their fellow citizens. This stigmatisation, related to, what Wittmer and Parizeau (2016: 92) describe as, waste picker’s ‘poverty, disorder and uncleanliness’, systematically undermines their legitimacy as frontline waste workers and has multiple ramifications for IWP’s lives and livelihoods. Our survey suggests that NMT is strongly linked to IWPs legitimisation efforts globally and has been shown to both lessen and contribute to the stigmatisation of workers. The bicycle presents a nuanced perspective within this context, embodying both positive and negative connotations. In Southern cities, it is often associated with poverty, while in rural areas, it carries a sense of prestige (Ardizzi et al., 2020; Rouse and Ali, 2002). Conversely, in industrialised countries, it is positively seen for its environmental and sporting merits. Nevertheless, cyclists are frequently perceived as an obstacle by users of conventional motorised transport (Steinmann, 2020). For recyclers, this discrimination is further reinforced by the stigma attached to their poverty and activity, therefore representing a phenomenon of intersectionality. In Vancouver, recyclers face constant harassment when riding (Elliott, 2019; Steinmann, 2020; Steinmann and Wilson, 2023). In one instance, a person was even told to ‘join the circus’ while riding a bicycle (Steinmann and Wilson, 2023: 208). Moreover, many recycling depots prohibit entry for individuals with shopping carts or bicycles, further exacerbating their marginalisation (Steinmann, 2020). Grocery carts, on the other hand, are always negatively connotated, as they are a symbol of homeless people and theft (Adeyemi, 2020; Chikowore and Kerr, 2020; Elliott, 2019; Gutberlet et al., 2009). IWPs using strollers are even more stigmatised as they are assumed to have stolen their vehicle from children, who are more vulnerable (Elliott, 2019).

Pushcarts, handcarts and animal carts are also clear social markers associated with poverty. In Brazil, the vehicles even gave their name to recyclers, pejoratively called *carroceiros* from *carroça* – horse-cart, or *carrinheiro* from *carrinho* – hand carts (Calafate-Faria, 2013; Melo, 2019). They are perceived as ‘subhuman scavenger and/or archaic intruders obstructing the flow of traffic’ (Melo, 2019: 153), are commonly called horses or donkeys as they pull carts (Coelho et al., 2019; de Andrade, 2022), and people avoid them by automatic fear of theft (de Andrade, 2022). In that context, having a bicycle is more accepted and recyclers are therefore socially more included than when using carts (Coelho et al., 2019). It was nonetheless reported that the design of a cart relates to reputation and can improve image and social acceptance (Bala et al., 2023; Brem et al., 2020; Gutberlet et al., 2009; Rouse and Ali, 2002).

#### Attachment and identity

The vehicle used by recyclers not only conveys a unique identity and social status but also carries a deep meaning and practical value that extends beyond the singular impression it may make on outsiders. Indeed, carts might provide shelter and a place to sleep (Calafate-Faria, 2013; Gutberlet et al., 2009). The bicycle may symbolise freedom (Steinmann, 2020; Steinmann and Wilson, 2022), social connection (Elliott, 2019; Steinmann and Wilson, 2022) and empowerment (Elliott, 2019), enabling them to maintain a routine (Steinmann and Wilson, 2022), access healthcare and employment (Steinmann and Wilson, 2023), potentially breaking the cycle of poverty (Steinmann and Wilson, 2022). It is worth noting that the level of attachment to these vehicles varies between recyclers, whereas some may feel little attachment due to frequent thefts (Steinmann, 2020), others take pride in their vehicles (Elliott, 2019). However, this last feeling does not extend to grocery carts, which do not have the same social significance (Elliott, 2019). Regardless of the type of vehicle, for many recyclers, it represents not only a long-standing aspect of their lives (Rouse and Ali, 2002; Steinmann, 2020) but also their most valuable possession (Rouse and Ali, 2002; Schenck and Blaauw, 2011). Furthermore, irrespective of its use, the vehicle remains an essential tool for recycling (Bala et al., 2023; Makina, 2020; Navarrete-Hernandez and Navarrete-Hernandez, 2018; Rouse and Ali, 2002; Steinmann and Wilson, 2022), facilitating transport (Calafate-Faria, 2013; Mitchell, 2008) and ensuring safety (Rouse and Ali, 2002). Therefore, acquiring a vehicle is often the priority for those entering the recycling profession, even if it means resorting to theft (Hayami et al., 2006; Schenck and Blaauw, 2011). Ultimately, despite the challenges highlighted, the necessity of non-motorised vehicles for their livelihoods overrides all else (Morais et al., 2022; Viljoen et al., 2018; Yu et al., 2020).

#### Gender and inequality

Gender relations and divisions among waste pickers remains under researched; however, the scholarship that does exist suggests waste picking activities are highly hierarchical, with gendered ideologies that perpetuate inequalities and enhance vulnerabilities for women (Dias and Ogando, 2015). According to our survey, NMT usage amongst IWPs reflects, and often perpetuates, pre-existing gender inequalities. Gender disparities linked to vehicle use vary with location, culture and religion. In some areas, women avoid street recycling because of safety concerns (Makina, 2020; Schenck and Blaauw, 2011) or unfair competition from men who can move faster and carry heavier loads (Bala et al., 2023; Rouse and Ali, 2002; Samson, 2020). Social norms further restrict women’s access to certain vehicles, such as the traditional Delhi dress, which excludes the use of bicycle carts (Rouse and Ali, 2002). Assumptions about women’s abilities can also hinder their access to better equipment. In Delhi, women were refused entry to a tricycle training programme (Amoah et al., 2023), whereas an NGO distributed carts with solid tyres to women and inflatable tyres to men, withholding the latter despite their superiority because of assumptions about women’s inability to repair punctures (Rouse and Ali, 2002). In addition, women’s limited ownership of vehicles exacerbates their dependency (Uteng, 2011). Furthermore, formalisation of the sector may reduce women’s employment opportunities, as men are hired for their strength (Wittmer, 2020). However, well-designed vehicles could help mitigate these inequalities (Rouse and Ali, 2002; Samson, 2020) and provide an entry point for women into a sector, which is, according to Bala et al. (2023) 75% male.

### Interventions

Although being denied legitimacy as the essential workers they are, there has been increased momentum globally towards supporting and integrating IWPs into waste management systems. Furthermore, and most importantly, waste pickers are, themselves, reclaiming their own legitimacy through organisation, activism, the professionalisation of their work. Given its importance in IWP lives and livelihoods, NMT has been a frequent space for intervention and innovation, with mixed success. For instance, many vehicles have been donated, repaired or stored by NGOs or cooperatives (Aparcana, 2017; Calafate-Faria, 2013; Coelho et al., 2019; Rouse and Ali, 2002; Wilson et al., 2009), which also offer education to the public to dispel stereotypes associated with certain vehicles (de Andrade, 2022; Gutberlet and Baeder, 2008). Community bike shops in Canada effectively assist recyclers by providing bikes and training in mechanics (Steinmann, 2020). The Binners Projects has designed a Universal Cart tailored for recycling (Binners’ Project, n.d.; Elliott, 2019), whereas the Urban Binning Unit has developed a quieter, more efficient trolley that enhances collection and transport while improving the social image of recyclers (Tremblay et al., 2010). In Victoria, a local engineer designs and distributes trolleys equipped to ease recyclers’ work and enhance their visibility, including a model with a built-in tent for shelter (Gutberlet et al., 2009). Pimp my Carroça (PMC), an NGO project, elevates the visibility and working conditions of recyclers by enhancing their carts with practical features or slogans like ‘don’t take our carts, learn how to recycle’ (de Andrade, 2022: 38). PMC also help recyclers in negotiating the return of confiscated vehicles and is expanding its advocacy efforts internationally through public events (de Andrade, 2022; Melo, 2019; Pimp My Carroça, n.d.).

Despite this, many of these organisations and political initiatives do not pay attention to the type of vehicle and its design, do not respond when maintenances need to be made (Dladla, 2018; Rouse and Ali, 2002), and consider unnecessary offers, such as adding number plates to carts (Calafate-Faria, 2013). Furthermore, reported failures include conflicts arising from projects distributing carts to new recyclers, leading to disputes over territory (Dladla, 2018).

## Discussion

The relationship between IWPs and NMT illustrates a dynamic nexus of environmental, social, economic, political and infrastructural challenges. This review shows that addressing these requires contextual sensitivity, interdisciplinary approaches and equitable stakeholder engagement. This discussion unpacks these interconnected dimensions, exploring implications for policy, design and future research.

Firstly, the relationship between IWPs and NMT is profoundly shaped by the specific geographic, infrastructural, social and economic contexts in which they operate. This review highlights the critical importance of tailoring interventions to local challenges. For instance, densely populated cities call for mobility and safety measures like dedicated cart lanes, whereas extreme weather conditions prioritise climate-appropriate cart designs. Gender disparities among IWPs also vary significantly by context. Women IWPs may face different barriers, including safety concerns, gender-based harassment and restricted access to transport options. Studies should aim to establish localised lists of priorities for IWPs, collaborating with stakeholders to co-design targeted interventions that are practical, inclusive and sustainable. Generic, top-down solutions often fail to address the nuanced realities faced by IWPs on the ground. Instead, interventions must be participatory and adaptable, reflecting the lived experiences of IWPs while fostering multi-stakeholder collaboration to create meaningful and lasting change.

Secondly, infrastructure plays a critical role in determining the safety, efficiency and dignity of IWPs. Poor urban design, insufficient policy support and exclusionary planning practices amplify risks and impose barriers that undermine IWPs’ livelihoods and health. For instance, IWPs navigating on traffic-dense city roads face higher exposure to accidents. The absence of protective infrastructure exacerbates physical harm. Poor air quality, unregulated waste transport and physically demanding cart designs contribute to long-term health issues such as respiratory diseases and musculoskeletal disorders. Badly designed or neglected infrastructure sends implicit messages about the value – or lack thereof – of IWPs to society. Substandard working environments perpetuate the stigmatisation of IWPs, reinforcing their marginalisation. Investment in infrastructure is not merely about enabling NMT but about affirming the human dignity of IWPs. Adequate parking, accessible repair facilities and formal routes for reclaimers should be prioritised to ensure safety, reduce stress and integrate IWPs into urban systems without compromising their autonomy. Cities that neglect these needs not only impose unnecessary risks on IWPs but also fail to recognise the broader economic and ecological value of their work.

Third, IWPs often face marginalisation due to the perception of their informality, heavily influenced by visual and social markers. Unlike formal municipal waste workers, who convey legitimacy through personal protective equipment, uniforms and official identification, IWPs are frequently stigmatised for their appearance and perceived vulnerability (Bermudez et al., 2019; Elliott, 2019; Shankar and Sahni, 2017). Poorly maintained carts, mismatched equipment and the absence of identification further reinforce negative public attitudes and create barriers to social and professional inclusion. Given that their mode of transport – often rudimentary, handmade or poorly maintained – is a highly visible component of their work, addressing the aesthetics of perceived formality in NMT presents a unique opportunity for reducing stigma and fostering greater inclusion. By improving the appearance and functionality of NMT, IWPs can not only gain legitimacy in the eyes of the public but also enhance their safety, efficiency and comfort. Programmes like *Pimp My Carroça* in Brazil have demonstrated the transformative potential of simple, targeted upgrades (Pimp My Carroça, n.d.). Repainting carts, adding logos and customising equipment can significantly enhance the visibility and legitimacy of IWPs, leading to better public reception and a sense of pride among participants. However, while these interventions show promise, there is limited empirical evidence on their broader impacts. Future research should examine how these aesthetic improvements intersect with functionality, safety and access to transport infrastructure. For instance, how do branded carts impact interactions with municipal workers or law enforcement? Do improved carts increase the efficiency of waste collection, or are these primarily social benefits? By focusing on NMT as both a tool of utility and a means of altering perceptions, research can provide critical insights into the role of transport in reducing stigma, improving livelihoods and integrating IWPs into formal systems. This dual focus on the aesthetics and functionality of NMT can bridge the gap between informal and formal waste sectors, while also informing participatory and context-sensitive interventions.

Fourthly, while top-down interventions are often misaligned with the realities of IWPs, community-driven initiatives have demonstrated the power of grassroots action. IWPs themselves are often best positioned to identify and address their needs. Reclaimers in many regions have independently adapted vehicles to suit their specific challenges, optimising for load capacity, durability and local terrain. Recognising the expertise and agency of IWPs is essential to creating interventions that are both effective and equitable. To build on this, policymakers and NGOs should prioritise partnerships with IWPs to co-design transport solutions that reflect their lived experiences and address their unique challenges. Research should similarly focus on capturing and leveraging the expertise of IWPs, exploring their innovations, preferences and practical knowledge about NMT and surrounding infrastructures. Such participatory research can ensure that transport interventions not only enhance the functionality and efficiency of their vehicles but also align with the broader social, cultural and economic contexts in which IWPs operate.

Finally, much has been said about the inequalities and challenges associated with NMT used by IWPs, but far less attention has been given to the significant positive impacts they achieve through its use. Their reliance on NMT not only facilitates environmental benefits but also generates economic and social contributions, yet the extent of these impacts remains largely unquantified. For instance, IWPs collect more than half of the waste in some cities (Uteng, 2011), and they do so without burning fossil fuels for mobility, thereby reducing the greenhouse gas emissions associated with waste collection and recycling (Sing, 2018). However, the precise scale of these contributions remains unclear and demands further exploration. Their presence is essential to the waste management ecosystem, and their NMT is a critical enabler of their work. For example, banning the use of donkey carts in Karachi is said to lead to uncollected waste piling up on the streets (Shah et al., 2019), highlighting the indispensable role of IWPs and their transport modes. Beyond practical waste collection, IWPs serve as agents of social, economic and environmental change. Through their use of NMT, they reduce urban waste, foster recycling and contribute to cleaner environments, all while supporting their own livelihoods and communities. Despite these significant contributions, the lack of recognition and understanding of the positive impacts IWPs achieve through NMT perpetuates their marginalisation. There is a need for research to quantify and highlight these impacts, environmental and economic, to ensure that their work and reliance on NMT are valued and integrated into broader waste management and urban development strategies.

Overall, despite the multi-disciplinary nature of NMT research, our understanding of its impact on IWPs remains limited. The existing literature is dominated by qualitative studies that explore the lived experiences of IWPs but lack rigorous quantitative analyses to substantiate key assumptions. Although it is presumed that NMT enhances livelihoods, there is little data on how infrastructure improvements could optimise economic outcomes. There is a dearth of studies quantifying the long-term health effects of NMT-related activities, despite some evidence of musculoskeletal issues and respiratory problems. The impacts IWPs achieve through their use of NMT remain unquantified in existing research. Most studies are region-specific, with little effort to synthesise findings across diverse geographies. A multi-lingual synthesis of existing literature would provide valuable insights and enable cross-regional comparisons.

## Conclusion

In conclusion, this mini-review sheds light on the multifaceted role of NMT in the lives of IWPs and the intricate challenges they face. The scarcity of peer-reviewed articles and quantitative studies points to a notable research deficiency that must be addressed to better inform policy and practice. The findings highlight the critical role of well-designed vehicles in enhancing the health, safety and livelihoods of IWPs, as well as the urgent need for supporting infrastructure – such as designated parking, maintenance services and protected routes. Moreover, the social significance of NMT cannot be overstated, as it reflects both the socio-economic status of recyclers and their sense of identity and empowerment. Although successful interventions associated with NMT often arise from grassroots projects, highlighting the importance of community-driven initiatives, this must go together with systemic changes and formalisation of the sector to tackle underlying structural issues. Nevertheless, policies frequently disregard the importance of IWPs’ vehicles, overlooking them in infrastructure planning. The review emphasises the necessity for further research to address gaps, particularly concerning NMT design, surrounding infrastructure, social status, as well as vehicle certifications and legal recognition of IWPs’ vehicles in public spaces. Cross-regional and multilingual studies should also be done to further understand the complexity of the subject. By addressing these complex challenges at the crossroads of health, social dynamics, urban policies and gender dynamics, society can strive towards a more just and inclusive future for all recyclers.
